# Serial assessment of the novel biomarker MFGE8 in comparison with GFAP for predicting severity and outcome after traumatic brain injury

**DOI:** 10.3389/fneur.2026.1750940

**Published:** 2026-01-20

**Authors:** Mingang Zou, Zhiji Tang, Defang Luo, Jianshen Zhong, Qianliang Huang, Qiuhua Jiang, Tao Hong

**Affiliations:** 1 Department of Neurosurgery, The Affiliated Ganzhou Hospital, Jiangxi Medical College, Nanchang University, Ganzhou, Jiangxi, China; 2 Department of Laboratory Medicine, The Affiliated Ganzhou Hospital, Jiangxi Medical College, Nanchang University, Ganzhou, Jiangxi, China; 3 Department of Neurosurgery, The First Affiliated Hospital, Jiangxi Medical College, Nanchang University, Nanchang, Jiangxi, China

**Keywords:** biomarker, GFAP, MFGE8, prognosis, severity, traumatic brain injury

## Abstract

**Background:**

Early assessment of traumatic brain injury (TBI) severity and prognosis is critical for clinical diagnosis and treatment. However, existing biomarkers have limitations in terms of predictive accuracy and timeliness. This study aimed to investigate the dynamic changes of the novel biomarker milk fat globule-EGF factor 8 (MFGE8) following TBI and systematically evaluate its value in predicting TBI severity and clinical prognosis, in comparison to GFAP.

**Methods:**

This was a prospective, single-center observational study involving 70 patients with acute TBI and 33 healthy controls. Serum levels of MFGE8 and GFAP were quantitatively determined by ELISA at admission (within 12 h of injury) and at 24, 48, and 72 h after injury. TBI severity was graded by the Glasgow Coma Scale (GCS) at admission, and the Rotterdam CT score was used to evaluate radiological injury severity. Outcome measures included 30-day mortality and the Glasgow Outcome Scale-Extended (GOSE) score at 3 months. Longitudinal biomarker trajectories and their associations with clinical severity, radiological severity, and outcomes were analyzed using linear mixed-effects models (LMMs), with Spearman’s correlation and receiver operating characteristic (ROC) analyses performed as complementary assessments.

**Results:**

The levels of serum MFGE8 and GFAP in TBI patients at all time points were significantly higher than those in the control group (*p* < 0.001) and peaked 24 h post-injury. In addition, significant correlations were shown between the two biomarkers, as well as between each biomarker and the clinical and radiographic severity scores (all *p* < 0.001). LMMs demonstrated significant differences in biomarker concentrations across clinical severity, radiological severity, and outcome groups, with significant main effects of time and group and significant group × time interactions. Pairwise comparisons indicated that GFAP provided earlier separation of clinical severity at admission, whereas MFGE8 exhibited its strongest severity separation at 24 h post-injury, with similar time-dependent patterns observed for radiological severity. Both biomarkers were consistently higher in patients with unfavorable outcomes and death compared with favorable survivors at admission and 24 h post-injury. MFGE8 showed superior prognostic performance for 30-day mortality at 24 h (AUC = 0.895), while GFAP demonstrated strong early discriminatory ability at admission. The GFAP/MFGE8 ratio exhibited moderate prognostic value.

**Conclusion:**

Serum MFGE8 is a promising biomarker after TBI. It is closely related to the injury severity and clinical prognosis. Particularly, it demonstrates high prognostic predictive value within 24 h post-injury. Continuous monitoring of MFGE8 may aid in early risk stratification and prognostic assessment for TBI.

## Introduction

Traumatic brain injury (TBI) is one of the leading causes of death and long-term disability worldwide, imposing a heavy burden on society (1). The pathophysiology of TBI is complex. Following the primary mechanical injury, a series of secondary damages are triggered, including neuroinflammation and excitotoxicity (2, 3). This process can last from hours to days and greatly impacts neurological outcomes. Therefore, identifying biomarkers that can objectively and dynamically reflect the pathophysiological progression and prognosis of TBI is crucial for achieving precise disease assessment and intervention.

Accurate determination of initial brain injury following TBI is essential for establishing neurological prognosis and balancing the risks and benefits of treatment options. Currently, clinical evaluation of TBI mainly relies on the Glasgow Coma Scale (GCS) and computed tomography (CT) imaging. However, GCS scores may be affected by factors such as sedation, alcohol, or shock, while CT, although effective in identifying macrostructural damage, has limited sensitivity for diffuse axonal injury or early secondary damage (4–6). More importantly, the pathophysiological process of TBI is progressive, and accurately assessing and quantifying the severity of the initial and subsequent secondary damage remains challenging (7). Against this backdrop, serum biomarker detection has emerged as a promising supplementary approach due to its non-invasive and dynamic monitoring characteristics. Current research has established several biomarkers associated with TBI. For instance, glial fibrillary acidic protein (GFAP), as a specific marker of astrocyte injury, has been proven to effectively distinguish CT-positive lesions and correlate with injury severity (8, 9). However, there is still controversy over its prognostic predictive efficacy, and most studies have focused on the population with mild to moderate TBI (10–12). Other classic markers such as S100B have their clinical accuracy and practicality limited due to their short half-life and relatively insufficient central specificity (13). Ubiquitin C-terminal hydrolase L1 (UCH-L1), although neuronal specific, may yield false positives in orthopedic trauma and is also expressed in neurodegenerative diseases, which limits its diagnostic specificity (14, 15). Overall, existing biomarkers mainly reflect acute structural damage and have limited ability to identify the dynamic process of secondary injury that drives prognostic outcomes.

Milk fat globule-EGF factor 8 (MFGE8), also known as lactadherin, is a secreted glycoprotein that plays a critical role in apoptotic cell clearance and inflammatory regulation. In the central nervous system, MFGE8 is predominantly expressed by microglia, with lower levels also detected in astrocytes and neurons (16, 17). In animal models of TBI and subarachnoid hemorrhage, the expression of MFGE8 in the brain is significantly upregulated, and exogenous supplementation of MFGE8 has been confirmed to alleviate neuroinflammation and promote the recovery of neurological function (18, 19). Mechanistically, MFGE8 acts as a bridging molecule by binding phosphatidylserine exposed on apoptotic cells or pathological extracellular vesicles, while simultaneously interacting with integrin αvβ3 on phagocytes, thereby facilitating efficient efferocytosis and limiting the accumulation of pro-inflammatory mediators (20–22). In addition, MFGE8 may exert potential anti-apoptotic and neuroprotective effects through binding to integrin-β3 and activating downstream signaling pathways, including the FAK/PI3K/AKT axis (18). Collectively, these findings suggest that endogenous MFGE8 upregulation may represent a crucial compensatory repair response to neural injury. Unlike GFAP, which mainly reflects astrocytic injury, MFGE8 is more likely associated with immune-inflammatory regulatory processes mediated by microglia or macrophages (23). This biological distinction indicates that MFGE8 and GFAP may reflect different dimensions of the pathological process in TBI, offering complementary potential. However, there is still a lack of clinical transformation evidence of serum MFGE8 in human TBI, especially whether its predictive efficacy can match or be complementary to GFAP, which requires systematic studies.

To address these gaps, we hypothesized that serum MFGE8 levels after traumatic brain injury exhibit time-dependent changes, are associated with injury severity, and relate to short-term neurological outcomes. To test these hypotheses, serial blood samples were collected from patients with TBI, and MFGE8 was systematically compared with GFAP. Linear mixed-effects models (LMMs) were applied to characterize longitudinal trajectories and predefined between-group differences based on GCS and Rotterdam CT severity stratifications, as well as their associations with short-term neurological outcomes. In addition, time-specific exploratory ROC analyses were conducted to compare the discriminatory performance of MFGE8 and GFAP at different post-injury time points.

## Methods

### Study population

This study is a prospective, single-center, observational investigation. A total of 70 patients diagnosed with acute TBI and 33 healthy volunteers were recruited from The Affiliated Ganzhou Hospital, Jiangxi Medical College, Nanchang University, which served as the control group. Healthy volunteers were matched for age, gender, and the absence of neurological or other severe systemic diseases. The diagnosis of TBI was made according to the guidelines for the management of traumatic brain injury (24). Inclusion criteria included the following: (1) voluntary signing of informed consent; (2) age between 18 and 80 years; (3) first blood sample collection within 12 h post-injury; (4) confirmed diagnosis of TBI; (5) head CT scan performed within 24 h of injury to confirm primary and secondary brain injuries. Exclusion criteria included the following: (1) history of neurological diseases (such as stroke, epilepsy, Alzheimer’s disease, Parkinson’s disease); (2) concomitant severe infections, immune system disorders, or malignant tumors; (3) history of brain surgery or recent (within 6 months) head trauma; (4) severe multiple injuries (Abbreviated Injury Scale, AIS ≥ 3); (5) pregnant or breastfeeding women; (6) individuals deemed unsuitable for participation by the investigator. The study protocol was approved by the hospital’s Ethics Committee and registered with the China Clinical Trial Center (Registration number: ChiCTR2500101228). Written informed consent was obtained from all patients or their legal guardians. All patients received standardized treatment according to current international guidelines.

### Serum collection and analysis

Peripheral blood samples were collected at admission, and at 24, 48, and 72 h post-injury for TBI patients, as well as once for healthy volunteers. Serum separation tubes were used to collect the blood samples. After clotting at room temperature for 45 ± 15 min, the samples were centrifuged at 1,000 × g for 20 min at 4 °C. The supernatant serum was aliquoted and immediately stored at −80 °C. Serum levels of MFGE8 (E-EL-H2063) and GFAP (E-EL-H6093) were measured using a double-antibody sandwich ELISA (Elabscience Biotechnology Co., Ltd). The sensitivities of the ELISA kit for detecting MFGE8 and GFAP were 37.5 pg./mL and 9.38 pg./mL respectively, and the detection ranges were 62.5–4,000 pg./mL and 15.63–1,000 pg./mL, respectively. The two kits have excellent specificity for their respective markers and have minimal cross-reactions with similar molecules. The intra-group and inter-group coefficients of variation of the kits were both less than 10%, and the recovery rates ranged from 80 to 120%. All calibrators and samples were run in double copies to ensure the accuracy and reliability of the results.

### Data collection and assessment procedures

The clinical severity of TBI was classified by the GCS score at admission, defined as: mild (13–15), moderate (9–12), and severe (3–8). Radiological severity was analyzed according to the Rotterdam CT classification, which derives a total score based on the presence of brain injury, including basal cisterns, midline shift, epidural mass lesions, and intraventricular or subarachnoid hemorrhage (25). For the convenience of analysis, the total score of Rotterdam CT was processed by dichotomy: 1–3 points were regarded as mild to moderate injury, and 4–6 points were regarded as severe injury. The use of this dichotomy is very common in the clinical trials of TBI and has been applied in previous biomarker studies (26, 27). Demographics, cause of injury, time to first sampling, GCS score, loss of consciousness, pupillary reactions, multiple trauma, ISS score, and neurosurgical intervention were recorded. The Glasgow Outcome Scale-Extended (GOSE) was used to assess outcomes at 3 months via structured interviews conducted in outpatient settings or by telephone. The GOSE ranges from 1 to 8, with higher scores corresponding to better functional outcomes (28). For categorical analyses, outcomes were grouped as: favorable survival (GOSE 5–8), unfavorable survival (GOSE 2–4), and death (GOSE 1). Blind evaluation was adopted to minimize bias as much as possible. Scores were reviewed by an independent committee of three certified neuroradiologists or neurologists, and disagreements were resolved by a third blinded reviewer. The inter-rater consistency of imaging and prognosis assessment was evaluated using Cohen’s kappa statistics, with detailed results presented in Supplementary Table S1.

### Statistical analysis

Normality was assessed using the Kolmogorov–Smirnov test. Continuous variables with a normal distribution were expressed as mean ± standard deviation (mean ± SD), whereas non-normally distributed variables were summarized as median (interquartile range, IQR). Categorical variables were described as frequencies and percentages. Biomarker concentrations were log10-transformed [log10(x + 1)] prior to modeling and visualization. Longitudinal trajectories were analyzed using LMMs, with time, group, and their interaction specified as fixed effects, and subject-specific random intercepts included to account for within-subject dependency. An autoregressive covariance structure of order 1 [AR(1)] was selected based on the Akaike Information Criterion (AIC) under maximum likelihood estimation, and final models were refitted using restricted maximum likelihood (REML). Model assumptions were evaluated using residual-versus-fitted plots and quantile–quantile (Q–Q) plots. Significant interaction effects were further explored using *post hoc* comparisons of estimated marginal means (EMMs) at each time point with Bonferroni correction. LMMs were performed for: (1) overall time effect; (2) clinical severity (GCS mild/moderate/severe) × time; (3) mild-to-moderate (GCS 9–15) vs. severe (GCS 3–8) × time; (4) Rotterdam CT (1–3 vs. 4–6) × time; and (5) outcome groups based on 3-month GOSE (favorable alive, unfavorable alive, death) × time. Correlations between parameters were examined using Spearman’s rank correlation coefficient. Receiver operating characteristic (ROC) curves and the area under the curve (AUC) were used to assess discriminatory performance and were considered exploratory. AUC values were categorized as follows: 0.9–1.0 = excellent, 0.8–0.9 = good, 0.7–0.8 = fair, 0.6–0.7 = poor, and 0.5–0.6 = no value (29). All statistical tests were two-tailed, with *p*-value <0.05 considered statistically significant. We did the statistical analyses using SPSS Statistics 21.0, MedCalc 20.022, and R software (version 4.5.1).

## Results

### Demographic and clinical characteristics

We included a total of 70 TBI patients and 33 healthy volunteers. There were no significant differences in age and gender between the two groups. The demographic and clinical characteristics of the participants are shown in Table 1. The cohort were mainly male (58.6%), and the primary causes of injury were ground-level falls (48.6%) and traffic accidents (42.9%). Overall, 27.1% of patients had multiple injuries. The median time from injury to the first blood collection was 4 h. The TBI cohort covered all severity levels based on the GCS at admission: mild (*n* = 28, 40%), moderate (*n* = 22, 31.4%), and severe (*n* = 20, 28.6%). According to the Rotterdam CT classification, 58 patients (82.8%) had a score of ≥3. Furthermore, 35.7% of patients required neurosurgical intervention. Regarding prognosis, the 30-day mortality rate was 11.4%, and at 3-month follow-up, 28.6% of patients had unfavorable outcomes, while 71.4% had favorable outcomes.

**Table 1:** Characteristics of patients and healthy volunteers.

Characteristics	TBI patients (*n* = 70)	Healthy volunteers (*n* = 33)
Age, years, median (IQR)	60 (48.8–67)	56 (48–64.5)
Gender, *n* (%)		
Male	41 (58.6)	18
Female	29 (41.4)	15
Cause of injury, *n* (%)		
Fall from height	5 (7.1)	
Traffic accident	30 (42.9)	
Ground-level fall	34 (48.6)	
Struck by object	1 (1.4)	
Time to first sampling, hours, median (IQR)	4 (2–7)	
Severity, *n* (%)		
Mild (GCS, 13–15)	28 (40)	
Moderate (GCS, 9–12)	22 (31.4)	
Severe (GCS, 3–8)	20 (28.6)	
Loss of consciousness, *n* (%)	20 (28.6)	
Pupillary reactions, *n* (%)		
Present	55 (78.6)	
One absent	6 (8.6)	
Bilateral absent	9 (12.9)	
Multiple trauma, *n* (%)	19 (27.1)	
ISS, median (IQR)	14 (9–17)	
Rotterdam CT classification, *n* (%)		
1	1 (1.4)	
2	11 (15.7)	
3	25 (35.7)	
4	14 (20)	
5	12 (17.1)	
6	7 (10)	
Neurosurgical intervention, *n* (%)	25 (35.7)	
Outcome, *n* (%)		
Unfavorable (GOSE, 1–4)	20 (28.6)	
Favorable (GOSE, 5–8)	50 (71.4)	
Death (GOSE 1)	8 (11.4)	
Survival (GOSE 2–8)	62 (88.6)	

GOSE, Glasgow outcome scale-extended; ISS, Injury severity score.

### Temporal dynamics of serum biomarkers after TBI

**Figure 1:**
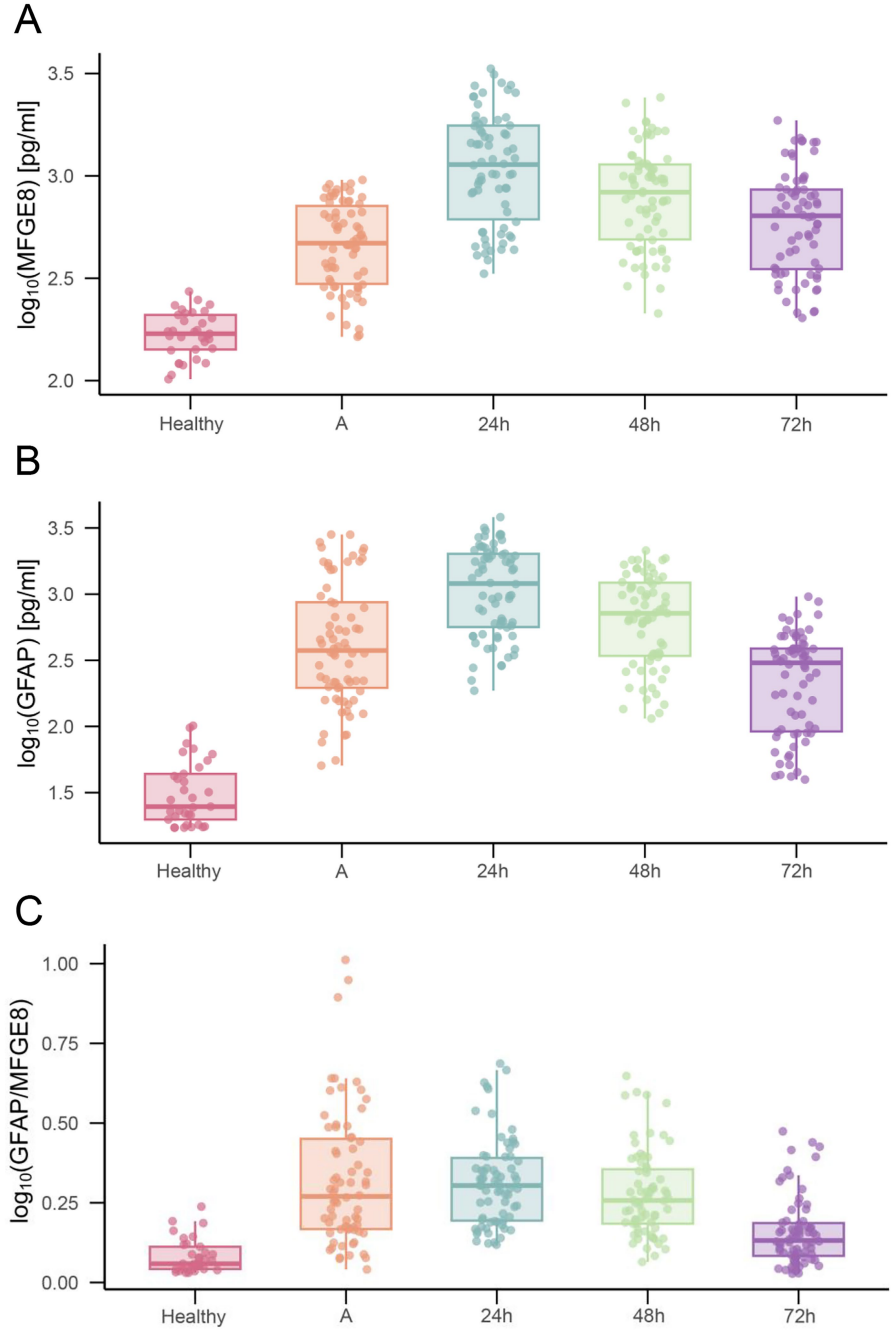
Serum biomarker concentrations over time after TBI. Serum levels of **(A)** MFGE8, **(B)** GFAP, and **(C)** the GFAP/MFGE8 ratio were measured in healthy controls and at admission, 24 h, 48 h, and 72 h after TBI. Data are presented as scatter-box plots (median with interquartile range) with log_10_-transformed concentrations.

The temporal profiles of serum MFGE8 and GFAP are shown in Figure 1. Compared with healthy volunteers, serum MFGE8 and GFAP concentrations in TBI patients were significantly elevated at admission (*p* < 0.001; Table 2). Descriptively, the median concentrations at admission were 468.33 pg./mL (IQR 291.99–731.37) for MFGE8 and 374.20 pg./mL (IQR 191.66–895.79) for GFAP. Within the TBI cohort, both MFGE8 and GFAP exhibited pronounced temporal changes. Serum concentrations increased from admission, reached their peak at 24 h post-injury (MFGE8: 1135.00 pg./mL, IQR 585.30–1767.70; GFAP: 1202.22 pg./mL, IQR 543.44–2032.96), and subsequently declined at 48 and 72 h. Despite this decrease, biomarker levels at 72 h remained significantly higher than those observed in healthy controls (both *p* < 0.001). Exploratory analysis of the GFAP/MFGE8 ratio revealed a similar temporal pattern, characterized by a peak at 24 h followed by a gradual decline (Figure 1C). LMMs confirmed a significant main effect of time for MFGE8 (*F* = 207.372, *p* < 0.001) and GFAP (*F* = 161.878, *p* < 0.001). Post-hoc comparisons based on EMMs showed that concentrations at 24 h were significantly higher than those at admission, 48 h, and 72 h for both biomarkers, and all pairwise comparisons between time points remained significant after Bonferroni adjustment (all adjusted *p* < 0.001). On the log10 scale, the estimated increase from admission to 24 h corresponded to an approximately 2.4-fold increase for MFGE8 and a 2.6-fold increase for GFAP.

**Table 2:** Serum MFGE8 and GFAP levels in TBI patients across serial time points and in healthy volunteers.

Biomarkers	Healthy (*n* = 33)	TBI (*n* = 70)			
		Admission (≤12 h)	24 h	48 h	72 h
MFGE8, pg./mL, median (IQR)	168.55 (140.33–210.41)	468.33 (291.99–731.37)	1135.00 (585.30–1767.70)	831.82 (476.88–1144.06)	637.67 (345.62–861.96)
GFAP, pg./mL, median (IQR)	23.79 (17.93–45.46)	374.20 (191.66–895.79)	1202.22 (543.44–2032.96)	715.38 (336.40–1235.76)	301.86 (89.41–393.86)

### Correlations of serum biomarkers with GCS scores and their interrelationship

Serum levels of MFGE8 and GFAP were significantly correlated with injury severity. MFGE8 exhibited a significant negative correlation with the GCS score at all time points (*p* < 0.001), with the strongest correlation observed at 24 h post-injury (*ρ* = −0.673; Table 3). MFGE8 also showed a significant positive correlation with the Rotterdam CT score at all time points (*p* < 0.01), again strongest at 24 h (ρ = 0.565; Supplementary Table S2). Similarly, GFAP demonstrated a significant negative correlation with the GCS score, strongest at admission (ρ = −0.644), and a significant positive correlation with the Rotterdam CT score, peaking at 24 h (ρ = 0.609). Furthermore, a significant positive correlation was observed between MFGE8 and GFAP levels across all time points (ρ range: 0.424–0.602; *p* < 0.001; Supplementary Table S3).

**Table 3:** Correlations between MFGE8, GFAP levels at sequential time points post-TBI and initial Glasgow Coma Scale scores.

Time point	MFGE8		GFAP	
	Spearman ρ	*P*	Spearman ρ	*P*
Admission	−0.539	<0.001	−0.644	<0.001
24 h	−0.673	<0.001	−0.635	<0.001
48 h	−0.487	<0.001	−0.537	<0.001
72 h	−0.407	<0.001	−0.454	<0.001

MFGE8, Milk fat globule-EGF factor 8; GFAP, Glial fibrillary acidic protein; TBI, Traumatic brain injury.

### Associations of serum biomarkers with TBI severity based on GCS scores

**Figure 2:**
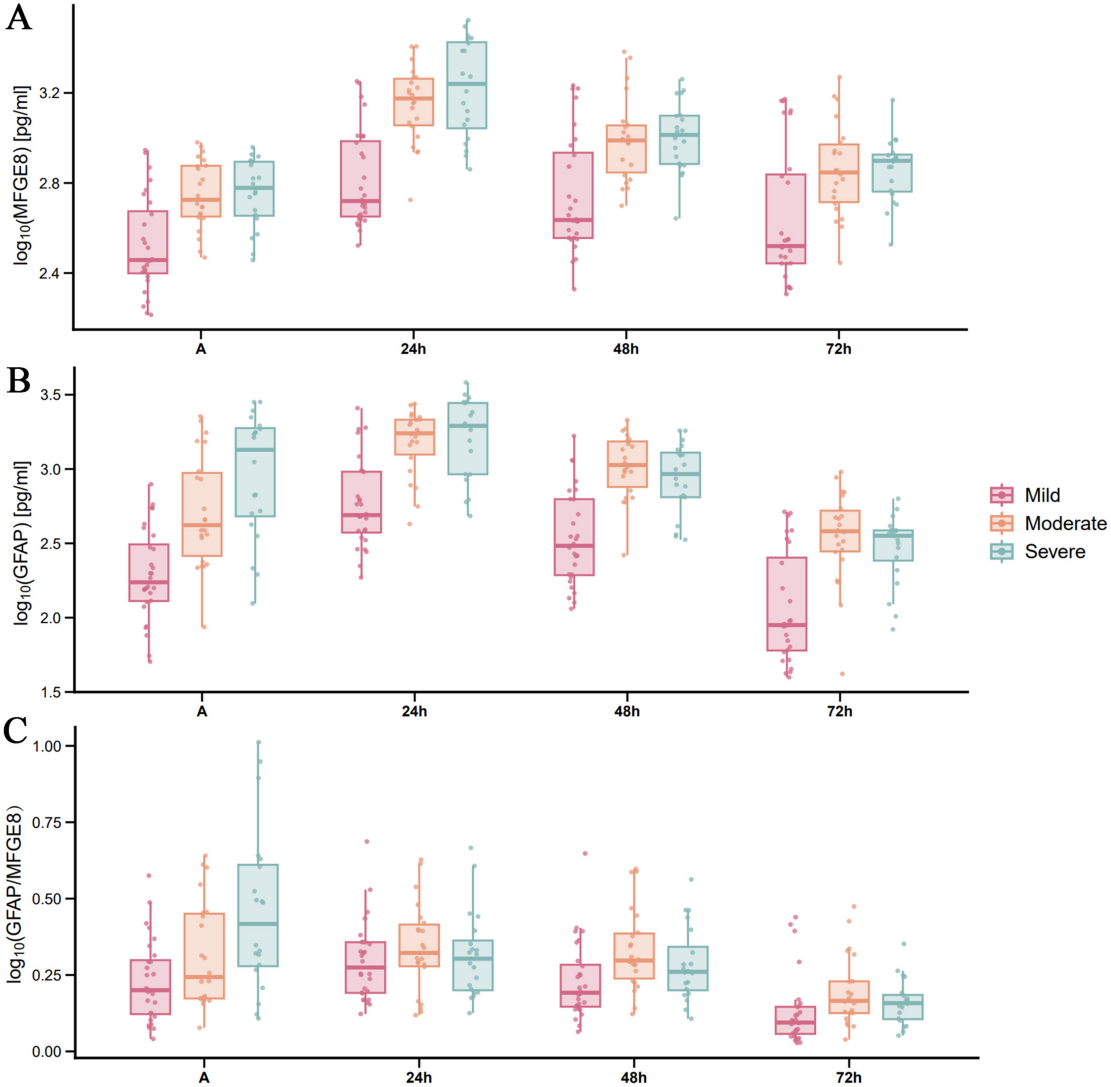
Temporal distribution of serum MFGE8 and GFAP levels, and the GFAP/MFGE8 ratio across TBI severity groups based on GCS scores. Serum levels of MFGE8, GFAP, and the GFAP/MFGE8 ratio in patients with **(A)** mild, **(B)** moderate, and **(C)** severe TBI (stratified by GCS) at admission and at 24, 48, and 72 h post-injury. Each row represents a distinct TBI severity group. Data are presented as scatter-box plots (median with interquartile range) with log_10_-transformed concentrations.

Figure 2 illustrates the dynamic changes in serum MFGE8 and GFAP levels, along with the GFAP/MFGE8 ratio, across TBI patients of varying severity. In general, the concentration of both biomarkers increased in a severity dependent manner and reached their peaks 24 h after injury. The log-transformed GFAP/MFGE8 ratio also showed a similar trend (median values: mild TBI group, 0.275; moderate TBI group, 0.323; severe TBI group, 0.304), and then gradually decreased (Figure 2C). LMMs demonstrated significant main effects of group and time on both MFGE8 and GFAP levels (all *p* < 0.001), as well as significant group × time interactions (MFGE8: *F* = 6.162, *p* < 0.001; GFAP: *F* = 3.519, *p* = 0.003). *Post hoc* analyses indicated that MFGE8 and GFAP concentrations were consistently higher in the moderate and severe TBI groups than in the mild group across most time points, whereas differences between the moderate and severe groups were not significant after Bonferroni adjustment (Supplementary Table S4). Given these findings and the clinical objective of distinguishing mild-to-moderate (GCS 9–15) from severe (GCS 3–8) TBI, additional binary-group LMMs were conducted and confirmed significant group × time interactions for both biomarkers (MFGE8: *F* = 7.178, *p* < 0.001; GFAP: *F* = 6.504, *p* < 0.001). Severe TBI was associated with higher MFGE8 and GFAP levels at early time points (e.g., MFGE8 at 24 h: estimate = −0.259, adj. *p* < 0.001; GFAP at 24 h: estimate = −0.266, adj. *p* = 0.008), with full time-specific contrasts provided in Supplementary Table S5. To complement the group-based analyses, exploratory ROC curve analyses were performed at selected time points (Supplementary Figure S1). MFGE8 showed its highest discriminative performance at 24 h (AUC = 0.766, 95% CI: 0.650–0.859), whereas GFAP performed best at admission (AUC = 0.788, 95% CI: 0.674–0.877). The GFAP/MFGE8 ratio demonstrated moderate discrimination at 24 h (AUC = 0.732, 95% CI: 0.613–0.831).

### Associations of serum biomarkers with TBI severity based on Rotterdam CT scores

When radiological severity was defined by admission Rotterdam CT scores (1–3 vs. 4–6), LMMs revealed significant main effects of group and time on both MFGE8 and GFAP levels (all *p* < 0.001), as well as significant group × time interactions (MFGE8: *F* = 7.282, *p* < 0.001; GFAP: *F* = 4.410, *p* = 0.005). *Post hoc* comparisons demonstrated that patients with higher Rotterdam CT scores (4–6) exhibited consistently higher MFGE8 and GFAP levels than those with lower scores (1–3) at all assessed time points. For MFGE8, the largest effect size was observed at 24 h post-injury (estimate = −0.306, adj. *p* < 0.001), whereas for GFAP, the strongest group difference occurred at admission (estimate = −0.489, adj. *p* < 0.001; Supplementary Table S6). Exploratory ROC analyses suggested consistent discrimination patterns across selected time points (Supplementary Figure S2). MFGE8 and GFAP showed AUC values greater than 0.70 at admission, 24 h, and 48 h, with MFGE8 reaching its highest AUC at 24 h (AUC = 0.808, 95% CI: 0.697–0.893). In contrast, the GFAP/MFGE8 ratio demonstrated only moderate discrimination at admission and limited discrimination at later time points.

### Association of serum biomarkers with clinical outcomes after TBI

**Figure 3:**
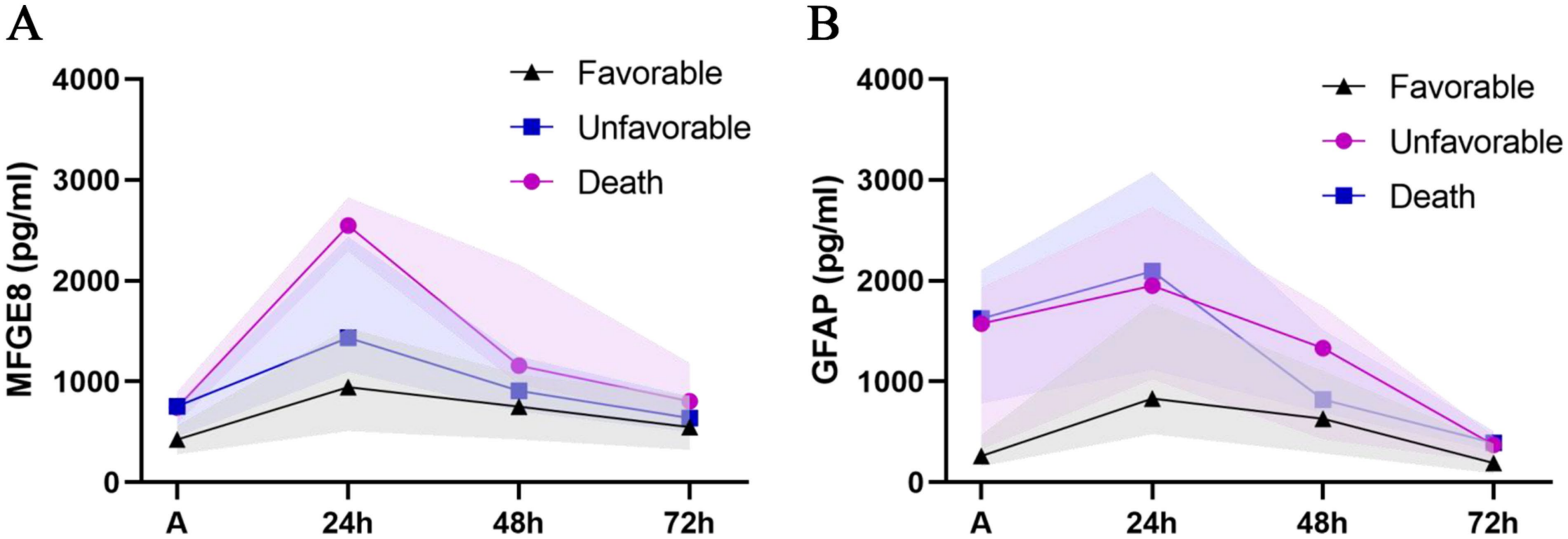
Temporal profiles of serum MFGE8 and GFAP levels stratified by clinical outcomes. Line charts depict the temporal trajectories of serum **(A)** MFGE8 and **(B)** GFAP concentrations stratified by clinical outcomes, categorized as favorable alive (GOSE 5–8), unfavorable alive (GOSE 2–4), and death (GOSE 1), measured at admission and at 24, 48, and 72 h after traumatic brain injury. Data points represent median values, and shaded areas indicate the interquartile range (IQR).

**Figure 4:**
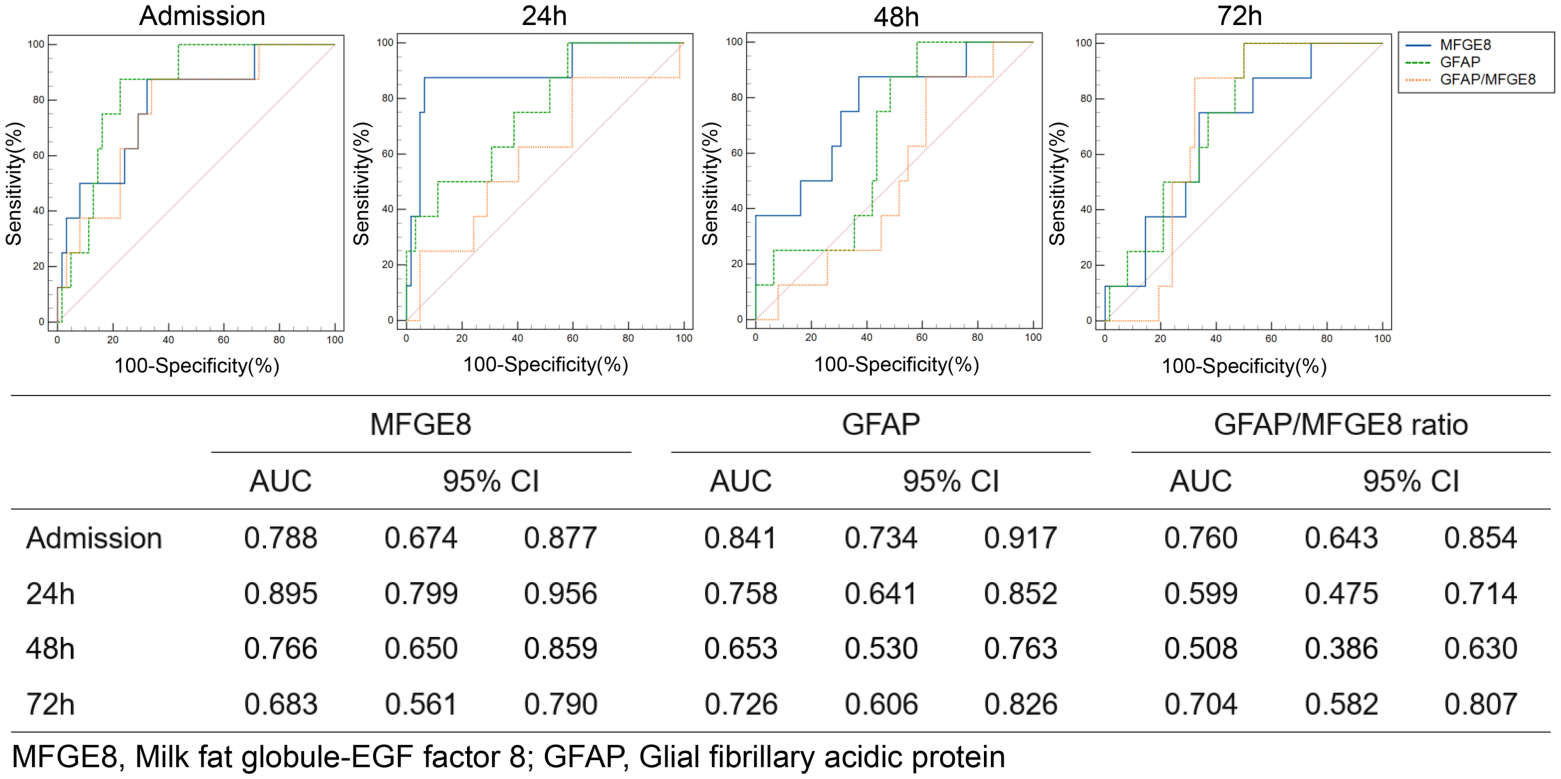
ROC curve analysis of MFGE8, GFAP, and the GFAP/MFGE8 ratio for predicting 30-day mortality risk.

Patients were categorized according to 3-month GOSE scores into three mutually exclusive outcome groups: favorable alive (GOSE 5–8), unfavorable alive (GOSE 2–4), and death (GOSE 1). The temporal profiles of serum MFGE8 and GFAP levels across outcome groups are shown in Figure 3. LMMs revealed significant main effects of outcome and time, as well as significant outcome × time interactions for both MFGE8 (outcome: *F* = 7.094, *p* = 0.002; time: *F* = 232.266, *p* < 0.001; interaction: *F* = 5.252, *p* < 0.001) and GFAP (outcome: *F* = 8.790, *p* < 0.001; time: *F* = 174.504, *p* < 0.001; interaction: *F* = 3.823, *p* = 0.002). *Post hoc* comparisons indicated that MFGE8 and GFAP levels were significantly higher in both the unfavorable alive and death groups than in the favorable alive group at early time points, particularly at admission and 24 h post-injury (adj. *p* < 0.05; Supplementary Table S7). In contrast, no significant differences were observed between the unfavorable alive and death groups at these time points. Differences between outcome groups attenuated at later time points, with only sporadic biomarker-specific contrasts remaining significant, consistent with the observed outcome × time interactions. Exploratory ROC analyses for 30-day mortality are presented in Figure 4, further illustrating the outcome-related trends observed in the LMM analyses. MFGE8 showed its highest AUC at 24 h (AUC = 0.895, 95% CI: 0.799–0.956), whereas GFAP showed higher AUC values at admission (AUC = 0.841, 95% CI: 0.734–0.917). The GFAP/MFGE8 ratio demonstrated moderate discrimination at admission (AUC = 0.760, 95% CI: 0.643–0.854). Exploratory ROC analyses for 3-month neurological outcomes are provided in Supplementary Figure S3. The overall results of the linear mixed-effects models assessing longitudinal associations of serum biomarkers with clinical severity, radiological severity, and outcomes are summarized in Table 4.

**Table 4:** Summary of linear mixed-effects models for longitudinal biomarker trajectories across clinical severity, radiological severity, and outcome groups.

Analysis (model)	Biomarker	Main effect of group		Main effect of time		Group × time interaction	
		*F* (*df*1, *df*2)	*p*	*F* (*df*1, *df*2)	*p*	*F* (*df*1, *df*2)	*p*
Clinical severity (GCS, 3-level)	MFGE8	15.284 (2, 67)	<0.001	241.019 (3, 201)	<0.001	6.162 (6, 201)	<0.001
	GFAP	26.245 (2, 67)	<0.001	172.345 (3, 201)	<0.001	3.519 (6, 201)	0.003
Clinical severity (GCS, binary)	MFGE8	7.511 (1, 68)	0.008	228.206 (3, 204)	<0.001	7.178 (3, 204)	<0.001
	GFAP	11.358 (1, 68)	0.001	173.886 (3, 204)	<0.001	6.504 (3, 204)	<0.001
Radiological severity (Rotterdam)	MFGE8	19.548 (1, 68)	<0.001	225.937 (3, 204)	<0.001	7.282 (3, 204)	<0.001
	GFAP	21.819 (1, 68)	<0.001	169.089 (3, 204)	<0.001	4.410 (3, 204)	0.005
Outcomes (GOSE)	MFGE8	7.094 (2, 67)	0.002	232.266 (3, 201)	<0.001	5.252 (6, 201)	<0.001
	GFAP	8.790 (2, 67)	<0.001	174.504 (3, 201)	<0.001	3.823 (6, 201)	0.002

Models were fitted separately for each grouping variable. Clinical severity was defined by admission GCS (3–8, 9–12, and 13–15; or binary 3–8 vs. 9–15), radiological severity by admission Rotterdam CT score (1–3 vs. 4–6), and outcomes by 3-month GOSE (favorable alive, GOSE 5–8; unfavorable alive, GOSE 2–4; death, GOSE 1).

## Discussion

This prospective study systematically compared the dynamic changes in the novel biomarker MFGE8 with the classic biomarker GFAP following TBI, using continuous-time point serological testing. It assessed their predictive value for injury severity and neurological outcomes. Longitudinal analyses based on LMMs demonstrated that serum MFGE8 levels significantly increased after TBI, peaking at approximately 24 h post-injury, and that the dynamic trajectory of MFGE8 was significantly associated with both clinical and radiological severity, as well as with 30-day mortality and 3-month neurological outcomes. These longitudinal associations were comparable to those observed for GFAP. Time-specific ROC analyses further supported the discriminatory ability of MFGE8 for neurological outcomes, with the most favorable performance observed at approximately 24 h post-injury. Collectively, these findings support MFGE8 as a TBI biomarker with considerable potential.

GFAP is a biomarker that has been approved by the U. S. Food and Drug Administration (FDA) in recent years for emergency screening for traumatic intracranial abnormalities (30). It also performs well in distinguishing TBIs of different severity (9). Therefore, this study selected GFAP as a reference for the value evaluation of MFGE8. TBI patients can be treated at different times after injury. When should these biomarkers be used for clinical assessment of TBI patients? First of all, the time distribution of GFAP and MFGE8 in serum should be clearly understood. In this study, the first blood sample was collected within 12 h after injury (median of 4 h), and the last sampling was 72 h after injury, which ensured the reliability of capturing the changes in the acute phase. As a cytoskeletal protein of astrocytes, GFAP levels increased significantly at admission and peaked at 24 h, which is consistent with previous studies (31, 32). GFAP concentration at admission showed the strongest correlation with the initial GCS score, consistent with its characteristics as a marker of acute structural injury. MFGE8, a multifunctional glycoprotein, plays an important role in central nervous system diseases by mediating the clearance of apoptotic cells and regulating neuroimmune inflammation. For instance, in neurodegenerative diseases, microglia clear damaged neurons and their fragments through MFGE8 to maintain the health of the neural network (33). In neuroinflammatory processes, MFGE8 can promote the clearance of apoptotic cells and reduce the inflammatory response by binding to phosphatidylserine and integrin receptors (34). This has also been confirmed in TBI and ischemic stroke models (18, 35). Given that neuroinflammation and apoptosis are core pathological processes in secondary brain injury after TBI, the dynamic changes of serum MFGE8 may reflect the biological functional state related to secondary brain injury after TBI to some extent. This study observed that MFGE8 and GFAP showed similar early elevation and peaking trends, and there was a moderate positive correlation between the two in the acute phase (Spearman’s *ρ* = 0.424–0.602). On one hand, this suggests that there may be a close link between astrocytic damage (GFAP release) and subsequently activated immunomodulatory response (MFGE8 level changes). On the other hand, it raises the question of the source of serum MFGE8. We speculate that its origin might be twofold. First, it may be directly released from central nervous system cells following blood–brain barrier (BBB) disruption. Second, it might originate from a systemic compensatory response to central nervous system injury. Some studies have found that neuroinflammatory responses can further damage BBB integrity, allowing central signals to enter the peripheral circulation (36). Therefore, the systemic response initiated by the body can be manifested as the compensatory secretion of serum MFGE8, which is likely to reflect the compensation of the need for central clearance.

Interestingly, the dynamic changes in the GFAP/MFGE8 ratio provide a new perspective for understanding the differences in their biological roles after TBI. The ratio peaked at 24 h post-injury and then continued to decline within 72 h. This time window is consistent with the critical period of secondary injury and the peak of death (37). The peak of this ratio may mark a turning point in the injury process. The difference in the ability of GFAP and MFGE8 to predict the severity of injury, namely that GFAP performs best at admission while MFGE8 does so at 24 h, further supports this possibility. GFAP can serve as a marker of early structural damage, while the peak level of MFGE8 more reflects the severity of secondary injury and the intensity of endogenous repair response. The continuous decline of the ratio after 24 h may mean that the repair mechanism gradually dominates. Therefore, continuous monitoring of the GFAP/MFGE8 ratio may provide a potential time window indicator for determining the pathological stage transition after TBI, which is conducive to more precise neuroprotective treatment intervention. This strategy is also consistent with the view of Thelin et al. (38), who argue that the combining biomarkers with different cellular origins and temporal dynamics can significantly improve predictive accuracy compared to an evaluation system relying on a single indicator.

The effectiveness of these biomarkers in distinguishing injury severity provides key insights for defining their clinical application scenarios. However, the severity assessment of TBI itself faces challenges: the accuracy of the initial GCS score of admission may be affected by factors such as sedation, poisoning or co-injury, and it may not match the degree of structural damage seen in imaging. Therefore, in this study, imaging severity defined by Rotterdam CT score was also included in addition to clinical GCS score to obtain more objective and complementary evidence of biological injury when assessing injury severity. We found that the concentration of both biomarkers increased in a severity dependent manner and reached their peaks 24 h after injury. In addition, GFAP could stably distinguish between mild-to-moderate and severe TBI patients, which is consistent with previous reports (9, 39). Notably, MFGE8 also showed this efficient dichotomous risk stratification ability, although both biomarkers could not further distinguish between moderate and severe injuries. This may reflect that when the damage exceeds a specific severity threshold, the release dynamics of biomarkers enter a high-level platform period, causing their concentration to no longer increase linearly with the intensification of clinical severity. Despite this limitation, this ability can still be used to clinically identify the most critical patients for rapid decision-making when traditional methods such as CT scan and GCS scores cannot provide an accurate assessment of the degree of injury. Additionally, the significant association between MFGE8 and the severity of imaging injuries further enhances its value as an objective biological indicator, providing an important basis for supplementing and calibrating the initial GCS assessment that may be interfered with, and thereby more reliably identifying potential severe structural injuries.

By repeatedly measuring GFAP and MFGE8 in the serum of the same patient cohort, we clarified the differences in their temporal spectrum for predicting prognosis in the acute phase of TBI. In this study, GFAP demonstrated moderate to good prognosis prediction ability in the early stage of admission, reflecting its clinical value in early risk assessment. This finding differs from the report by Huebschmann et al. (10). The difference may mainly come from the GCS distribution characteristics of the study population: in our cohort, the proportions of mild, moderate and severe TBI patients are relatively balanced (40, 31.4, and 28.6% respectively), while their study focused on a single mild group, which may lead to dilution or deviation in the predictive efficacy of GFAP. For MFGE8, the data showed that its serum level increased significantly overall in non-survivors and poor prognosis, demonstrating good prognostic discrimination efficacy. Importantly, MFGE8’s predictive ability at 24 h post-injury was better than that of GFAP at all time points, and it remained elevated at 48 h, thereby broadening the clinical monitoring window of TBI biomarkers. Therefore, we speculate that persistently high levels of MFGE8 may imply an unbalanced compensatory repair response: despite the initiation of repair processes in response to severe tissue damage, the response may not be enough to offset the continuous damage, which ultimately leads to the failure to restore neuroimmune homeostasis. This hypothesis is consistent with the temporal findings of this study: the continuous high expression of GFAP indicates the persistence of damage, while the correlation between the peak of MFGE8 and the poor prognosis suggests the failure of the repair mechanism. These findings indicate that MFGE8 is not only a potential prognostic biomarker, but also an important indicator for assessing the dynamic balance of damage and repair after TBI. In the future, multi-center and large-scale studies are needed to verify its clinical value, and to explore its specific mechanism in the pathological evolution of TBI with the help of basic experiments. In addition, established multimodal prognostic models for TBI, such as the IMPACT score, integrate neurological examination, CT findings, and laboratory variables; in this context, MFGE8 may provide complementary biological information related to secondary injury and repair, potentially enhancing the predictive performance of such models.

This study has several limitations. First, as a single-center observational study, the sample size is limited and the population source is relatively single, which may affect the universality of the conclusion. Secondly, there is a lack of long-term follow-up data on neurological function. It should be emphasized that MFGE8 is not a biomarker specific to the central nervous system. Although strict inclusion criteria were adopted (such as excluding patients with severe multiple trauma) and GFAP was used as a reference to indirectly evaluate the relative contribution of peripheral sources to the changes in MFGE8, background signals from the peripheral immune system or endothelial system could not be completely eliminated. Furthermore, all the tests were conducted on serum samples without simultaneous cerebrospinal fluid (CSF) testing, which limited the ability to directly explain specific changes in the central nervous system. Future research should focus on combining CSF testing to clarify the central specificity of MFGE8 and verify its clinical value in a larger population through long-term follow-up.

## Conclusion

This study identified the dynamic changes of the novel biomarker MFGE8 after TBI and emphasized its close correlation with the severity of injury and clinical outcomes, especially demonstrating the best prognostic predictive ability within 24 h after injury. Unlike GFAP, which serves as an early marker of structural damage, the temporal characteristics of MFGE8 suggest that it is more indicative of the secondary injury process following TBI. Thus, MFGE8 holds potential as a biomarker for assessing TBI severity and prognosis, providing valuable support for clinical grading and treatment decision-making.

## Data Availability

The original contributions presented in the study are included in the article/Supplementary material, further inquiries can be directed to the corresponding author.
