# Supplementary material for: Serial assessment of the novel biomarker MFGE8 in comparison with GFAP for predicting severity and outcome after traumatic brain injury
Source: Front Neurol. 2026 Jan 20;17:1750940. doi: 10.3389/fneur.2026.1750940 (PMC12864137; doi:10.3389/fneur.2026.1750940)
Supplement: Supplementary file 1 [file Supplementary_file_1.docx]

Supplementary Material

# Supplementary Figures and Tables

## Supplementary Tables

**Supplementary Table S1** Inter-rater reliability of imaging severity and outcome assessments.

| **Variable** | **Cohen’s κ** | **95% CI** | **Interpretation (Landis & Koch)** |
| --- | --- | --- | --- |
| Rotterdam CT score | 0.907 | 0.829 – 0.985 | Almost Perfect |
| GOSE | 0.845 | 0.753–0.937 | Almost Perfect |

**Supplementary Table S2** Correlations between MFGE8、GFAP levels at sequential time points post-TBI and initial Rotterdam CT scores.

|  | **MFGE8** | |  | **GFAP** | |
| --- | --- | --- | --- | --- | --- |
|  | Spearman ρ | *P* |  | Spearman ρ | *P* |
| Admission | 0.454 | ＜0.001 |  | 0.545 | ＜0.001 |
| 24h | 0.565 | ＜0.001 |  | 0.609 | ＜0.001 |
| 48h | 0.401 | 0.001 |  | 0.449 | ＜0.001 |
| 72h | 0.314 | 0.008 |  | 0.351 | 0.003 |

MFGE8, Milk fat globule-EGF factor 8; GFAP, Glial fibrillary acidic protein; TBI, Traumatic brain injury.

**Supplementary Table S3** Spearman correlation coefficients between MFGE8 and GFAP at different time points.

|  | **Rho** | ***P* Value** |
| --- | --- | --- |
| Admission | 0.456 | ＜0.001 |
| 24h | 0.602 | ＜0.001 |
| 48h | 0.509 | ＜0.001 |
| 72h | 0.424 | ＜0.001 |

Rho, Spearman correlation coefficients.

**Supplementary Table S4** Pairwise comparisons derived from LMMs for clinical severity (GCS: Mild, Moderate, and Severe groups).

| **Time point** | **Pairwise contrast** | **MFGE8** | |  | **GFAP** | |
| --- | --- | --- | --- | --- | --- | --- |
|  |  | Est. (95% CI) | Adj. *p* |  | Est. (95% CI) | Adj. *p* |
| Admission | Severe - Moderate | 0.006 (-0.151, 0.164) | 1.000 |  | 0.243 (0.006, 0.480) | 0.043 |
|  | Severe - Mild | 0.234 (0.085, 0.383) | <0.001 |  | 0.670 (0.445, 0.894) | <0.001 |
|  | Moderate - Mild | 0.228 (0.082, 0.373) | <0.001 |  | 0.427 (0.208, 0.646) | <0.001 |
| 24 h | Severe - Moderate | 0.072 (-0.086, 0.229) | 0.802 |  | 0.034 (-0.204, 0.271) | 1.000 |
|  | Severe - Mild | 0.407 (0.258, 0.556) | <0.001 |  | 0.448 (0.223, 0.673) | <0.001 |
|  | Moderate - Mild | 0.335 (0.190, 0.480) | <0.001 |  | 0.414 (0.196, 0.633) | <0.001 |
| 48h | Severe - Moderate | 0.011 (-0.147, 0.168) | 1.000 |  | -0.083 (-0.320, 0.155) | 1.000 |
|  | Severe - Mild | 0.262 (0.113, 0.411) | <0.001 |  | 0.405 (0.180, 0.630) | <0.001 |
|  | Moderate - Mild | 0.251 (0.106, 0.396) | <0.001 |  | 0.488 (0.269, 0.706) | <0.001 |
| 72h | Severe - Moderate | 0.001 (-0.157, 0.158) | 1.000 |  | -0.082 (-0.319, 0.155) | 1.000 |
|  | Severe - Mild | 0.213 (0.064, 0.362) | 0.003 |  | 0.404 (0.179, 0.628) | <0.001 |
|  | Moderate - Mild | 0.212 (0.067, 0.357) | 0.002 |  | 0.485 (0.267, 0.704) | <0.001 |

Estimates (Est.) represent pairwise differences in model-based EMMs on the log10(x + 1) scale, calculated as first group minus second group as indicated in the contrast column. Negative values indicate higher concentrations in the second group.

**Supplementary Table S5** Pairwise comparisons derived from LMMs for clinical severity (GCS: Mild-Moderate vs. Severe).

| **Time point** | **MFGE8** | |  | **GFAP** | |
| --- | --- | --- | --- | --- | --- |
|  | Est. (95% CI) | Adj. *p* |  | Est. (95% CI) | Adj. *p* |
| Admission | -0.134 (-0.257, -0.011) | 0.033 |  | -0.482 (-0.675, -0.288) | <0.001 |
| 24 h | -0.259 (-0.382, -0.137) | <0.001 |  | -0.266 (-0.459, -0.072) | 0.008 |
| 48h | -0.152 (-0.274, -0.029) | 0.016 |  | -0.190 (-0.384, 0.003) | 0.054 |
| 72h | -0.120 (-0.242, 0.003) | 0.056 |  | -0.190 (-0.384, 0.004) | 0.054 |

Est. (95% CI) is the difference in EMMs for log10(x+1)-transformed concentrations, calculated as (Mild–Moderate) − (Severe) at each time point. Negative values indicate higher levels in the severe group.

**Supplementary Table S6** Pairwise comparisons derived from LMMs for radiological severity (Rotterdam CT: 1-3 vs. 4-6).

| **Time point** | **MFGE8** | |  | **GFAP** | |
| --- | --- | --- | --- | --- | --- |
|  | Est. (95% CI) | Adj. *p* |  | Est. (95% CI) | Adj. *p* |
| Admission | -0.198 (-0.303, -0.094) | <0.001 |  | -0.489 (-0.657, -0.322) | <0.001 |
| 24 h | -0.306 (-0.410, -0.201) | <0.001 |  | -0.333 (-0.501, -0.166) | <0.001 |
| 48h | -0.218 (-0.322, -0.114) | <0.001 |  | -0.268 (-0.436, -0.101) | 0.002 |
| 72h | -0.164 (-0.268, -0.059) | 0.003 |  | -0.262 (-0.429, -0.094) | 0.003 |

Est. (95% CI) is the difference in estimated marginal means for log10(x+1)-transformed concentrations, calculated as (Rotterdam 1-3) − (Rotterdam 4-6) at each time point. Negative values indicate higher biomarker levels in the Rotterdam 4-6 group.

**Supplementary Table S7** Pairwise comparisons across 3-month outcome groups (GOSE: Favorable vs. Unfavorable vs. Death).

| **Time point** | **Pairwise contrast** | **MFGE8** | |  | **GFAP** | |
| --- | --- | --- | --- | --- | --- | --- |
|  |  | Est. (95% CI) | Adj. *p* |  | Est. (95% CI) | Adj. *p* |
| Admission | Favorable - Unfavorable | -0.191 (-0.364, -0.018) | 0.028 |  | -0.452 (-0.728, -0.176) | <0.001 |
|  | Favorable - Death | -0.230 (-0.436, -0.025) | 0.024 |  | -0.655 (-0.982, -0.328) | <0.001 |
|  | Unfavorable - Death | -0.039 (-0.286, 0.207) | 0.922 |  | -0.203 (-0.594, 0.190) | 0.435 |
| 24 h | Favorable - Unfavorable | -0.236 (-0.409, -0.063) | 0.005 |  | -0.283 (-0.559, -0.007) | 0.043 |
|  | Favorable - Death | -0.416 (-0.621, -0.211) | <0.001 |  | -0.346 (-0.673, -0.019) | 0.036 |
|  | Unfavorable - Death | -0.180 (-0.426, 0.066) | 0.193 |  | -0.063 (-0.455, 0.329) | 0.922 |
| 48h | Favorable - Unfavorable | -0.144 (-0.318, 0.029) | 0.121 |  | -0.208 (-0.484, 0.068) | 0.176 |
|  | Favorable - Death | -0.253 (-0.458, -0.047) | 0.012 |  | -0.248 (-0.575, 0.079) | 0.173 |
|  | Unfavorable - Death | -0.108 (-0.354, 0.138) | 0.545 |  | -0.040 (-0.432, 0.352) | 0.968 |
| 72h | Favorable - Unfavorable | -0.103 (-0.276, 0.070) | 0.335 |  | -0.192 (-0.468, 0.085) | 0.227 |
|  | Favorable - Death | -0.180 (-0.386, 0.025) | 0.096 |  | -0.358 (-0.685, -0.031) | 0.029 |
|  | Unfavorable - Death | -0.078 (-0.324, 0.169) | 0.732 |  | -0.167 (-0.559, 0.225) | 0.568 |

Estimates (Est.) are pairwise differences of model-based estimated marginal means on the log10(x+1) scale, calculated in the order shown in the contrast column. 95% CI, confidence interval. P values were adjusted using Bonferroni correction within each time point.

## Supplementary Figures

**Supplementary Figure 1** ROC curve analysis of MFGE8, GFAP, and the GFAP/MFGE8 ratio for discriminating between mild-to-moderate and severe TBI.

**Supplementary Figure 2** ROC curve analysis of MFGE8, GFAP, and the GFAP/MFGE8 ratio for differentiating TBI severity based on the Rotterdam CT classification.

**Supplementary Figure 3** ROC curve analysis of MFGE8, GFAP, and the GFAP/MFGE8 ratio for predicting 3-month neurological outcomes.
